# Supplementary material for: Human altruistic tendencies vary with both the costliness of selfless acts and socioeconomic status
Source: PeerJ. 2016 Oct 27;4:e2610. doi: 10.7717/peerj.2610 (PMC5088574; doi:10.7717/peerj.2610)
Supplement: File S1 — Summary data by suburb with total number of returned letters (10 stamped and 10 unstamped letters dropped per suburb), Socioeconomic Index, and number of postboxes. [file peerj-04-2610-s001.docx]

**Supplemental File 1**

Summary data by suburb with total number of returned letters (10 stamped and 10 unstamped letters dropped per suburb), Socioeconomic Index, and number of postboxes.

| **Suburb** | **Socioeconomic**  **Index** | **# Postboxes** | **# Letters Returned** | |
| --- | --- | --- | --- | --- |
|  |  |  | **Stamped** | **Unstamped** |
| Medina | 1 | 1 | 3 | 4 |
| Kwinana | 2 | 1 | 5 | 1 |
| Hillman | 3 | 0 | 7 | 3 |
| Coolbellup | 3 | 1 | 3 | 2 |
| Warnbro | 4 | 1 | 4 | 1 |
| Rivervale | 5 | 3 | 5 | 2 |
| Willagee | 5 | 1 | 5 | 3 |
| Kallaroo | 6 | 3 | 9 | 3 |
| Madeley | 7 | 1 | 7 | 2 |
| Kingsley | 8 | 3 | 4 | 5 |
| Nedlands | 9 | 10 | 8 | 3 |
| Cottesloe | 9 | 8 | 6 | 4 |
| Dalkeith | 10 | 5 | 10 | 3 |
| City Beach | 10 | 5 | 8 | 5 |
| Peppermint Grove | 10 | 0 | 8 | 4 |
